# Use and safety of magnetic resonance imaging in patients with an implanted MRI-conditional pacing device

**DOI:** 10.1186/1532-429X-18-S1-P199

**Published:** 2016-01-27

**Authors:** Maurits van der Graaf, Pranav Bhagirath, Marco Götte

**Affiliations:** Cardiology, Haga Teaching Hospital, Den Haag, Netherlands

## Background

This study investigated the use and safety of Magnetic Resonance Imaging (MRI) for several pathologies in patients with an implanted MRI conditional pacing system.

## Methods

This was a retrospective, single center study. All patients with an MRI conditional pacemaker or Internal Cardioverter Defibrillator (ICD) implanted in our center between June 2011 and March 2015 were enrolled. All MRI examinations performed in these patients were evaluated. For all patients, pacing thresholds, P- and R-wave amplitude, lead impedance and battery longevity were monitored during the entire follow-up.

## Results

**Figure 1:**
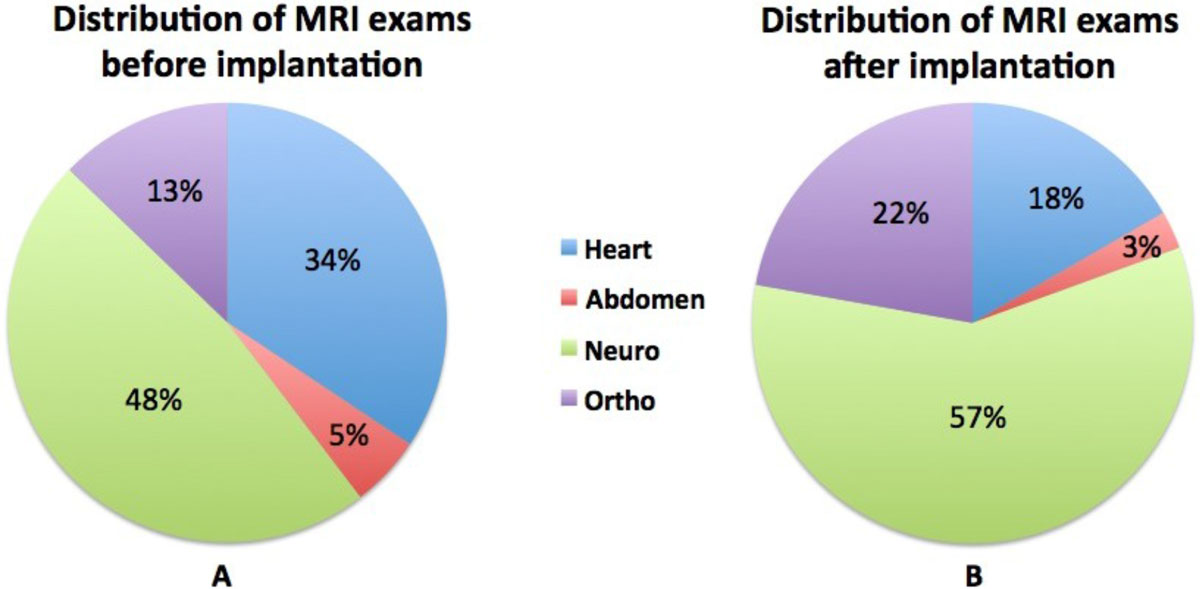
Figure 1

In total, 214 patients with an implanted MRI conditional device (mean age 65 ± 14; 64% male; 138 pacemakers; 76 ICDs) were enrolled. At the time of implant, 94% of these patients suffered from a co-morbidity that may lead to a future indication for MRI. In 24 patients (11%), 36 MRI investigations were safely performed during a mean follow-up period of 406 days (51-1353 days). The majority of the scans were performed to confirm, exclude or follow-up on neurological disease (figure 1). There were no significant changes in lead impedance or sensing threshold between the pre- and post-procedural measurements.

## Conclusions

Since MRI conditional devices are not yet available for all patients, patients that have a high likelihood of requiring an MRI examination in the near future need to be selected before implantation of these devices.

